# Supplementary material for: Pemafibrate Protects against Fatty Acid-Induced Nephropathy by Maintaining Renal Fatty Acid Metabolism
Source: Metabolites. 2021 Jun 9;11(6):372. doi: 10.3390/metabo11060372 (PMC8230306; doi:10.3390/metabo11060372)
Supplement: Supplementary file 1 [file metabolites-11-00372-s001.zip › metabolites-1214372-supplementary.pdf]

metabolites

MDPI

Supplementary Materials

Supplementary table S1. Results of blood tests in experiment 1.

	TP (g/dL)	ALB (g/dL)	ALT (IU/L)	TG (mg/dL)	HDL (mg/dL)	LDL (mg/dL)	BUN (mg/dL)	Cr (mg/dL)	FFA (mEq/L)
Veh	5.20 (±0.12)	0.60 (±0.03)	35.4 (±4.02)	99.9 (±12.8)	57.5 (±2.68)	5.36 (±0.30)	31.1 (±0.89)	0.10 (±0.01)	0.86 (±0.13)
PEM	5.23 (±0.07)	0.64 (±0.02)	41.9 (±4.48)	41.0 (±4.15) #	70.1 (±2.09) #	4.79 (±0.29)	35.1 (±1.22) #	0.10 (±0.00)	0.78 (±0.11)

Significant differences with the Veh group are indicated with # ($P < 0.05$). Abbreviations: TP, total protein; ALB, albumin; ALT, alanine aminotransferase; TG, serum triglyceride; HDL, high-density lipoprotein; LDL, low-density lipoprotein; BUN, blood urea nitrogen; Cr, creatinine; FFA, free fatty acid.

Supplementary figure S1. Other results in experiment 1.

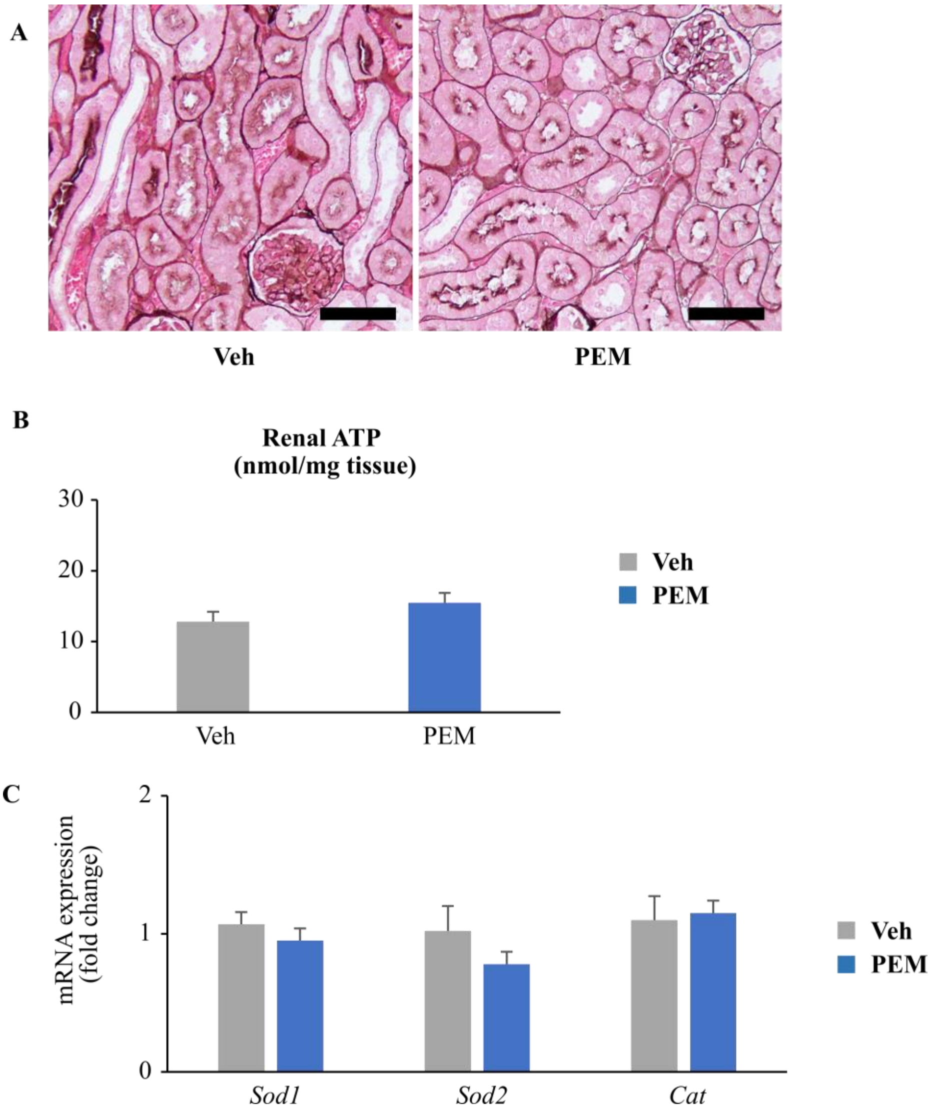

Other results in experiment 1. (A) Light microscopic analysis of tubular lesions. The sections were stained with periodic acid-methenamine-silver. Scale bar = 100 µm. (B) Renal adenosine triphosphate content. (C) Renal expressions of mRNA related to antioxidant agents. In those analysis, any obvious or significant differences was not detected between the Veh and PEM group.

Supplementary table S2. Results of blood tests in experiment 2.

	TP (g/dL)	ALB (g/dL)	ALT (IU/L)	TG (mg/dL)	HDL (mg/dL)	LDL (mg/dL)
CON	4.98 (±0.11) #	0.53 (±0.02) #	32.0 (±4.86) #	110 (±12.1)	59.9 (±2.39) #	8.79 (±1.97) #
FAON	10.6 (±0.39)	1.51 (±0.07)	13.3 (±2.06)	80.3 (±14.3)	16.4 (±1.94)	2.62 (±0.51)
PEM + FAON	10.2 (±0.40)	1.50 (±0.08)	15.8 (±4.66)	34.9 (±10.6) #	22.3 (±3.00)	1.50 (±0.17)

Significant differences with the FAON group are indicated with # ($P < 0.05$). Abbreviations: TP, total protein; ALB, albumin; ALT, alanine aminotransferase; TG, serum triglyceride; HDL, high-density lipoprotein; LDL, low-density lipoprotein.

Supplementary figure S2. Pathological findings in glomeruli in experiment 2.

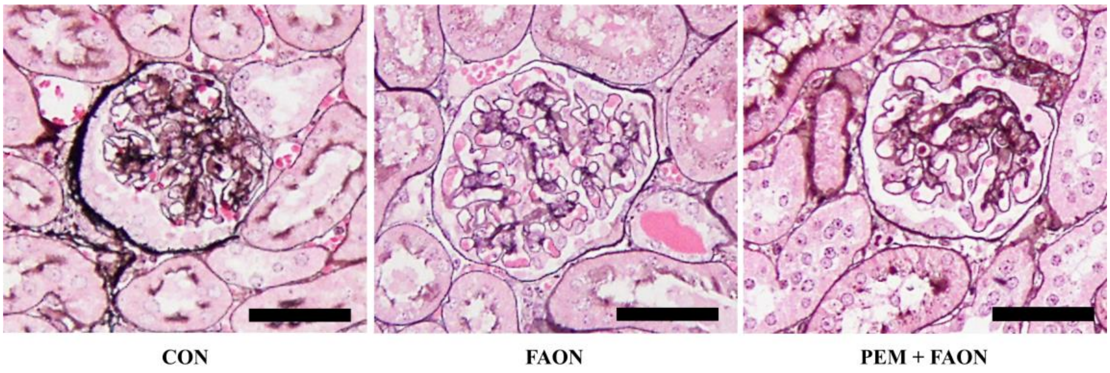

Light microscopic analysis of glomeruli. The sections were stained with periodic acid-methenamine-silver. Scale bar = 100 μm.

Supplementary table S3. Primers used in mRNA analyses.

Gene			Primer		GenBank accession no.
Ppara	Forward	5'-	CCTCAGGGTACCACTACGGAGT	-3'	NM_011144
	Reverse	5'-	GCCGAATAGTTCGCCGAA	-3'	
Cpt2	Forward	5'-	ATCGTACCCACCATGCACTAC	-3'	NM_009949
	Reverse	5'-	CTGTCATTCAAGAGAGGCTTCTG	-3'	
Acadvl	Forward	5'-	GCGTGTGCTCCGAGATATTC	-3'	NM_017366
	Reverse	5'-	CCAGTGAGTTCCTTTCCTTTG	-3'	
Acadm	Forward	5'-	TGCTTTTGATAGAACCAGACCTACAGT	-3'	NM_007382
	Reverse	5'-	CTTGGTGCTCCACTAGCAGCTT	-3'	
Hadha	Forward	5'-	CCTTTATCCTGCCCCTTTG	-3'	NM_178878
	Reverse	5'-	GCGATTCAGCAAGATAACCA	-3'	
Acox1	Forward	5'-	TGGTATGGTGTCGTACTTGAATGAC	-3'	NM_015729
	Reverse	5'-	AATTTCTACCAATCTGGCTGCAC	-3'	
Ehhadh	Forward	5'-	CGATACTCTTCCCCCACTACCA	-3'	NM_023737
	Reverse	5'-	CAGTTACCAACAACGACTCCAATC	-3'	
Acaa1	Forward	5'-	TCTACGGTCAACAGACAGTGTTCA	-3'	NM_146230
	Reverse	5'-	GGCCATGCCAATGTCATAAGA	-3'	
Sod1	Forward	5'-	AAGCGGTGAACCAGTTGTGTT	-3'	NM_011434
	Reverse	5'-	AGCCTTGTGTATTGTCCCCATACT	-3'	
Sod2	Forward	5'-	TCCCAGACCTGCCTTACGACTAT	-3'	NM_013671
	Reverse	5'-	GGTGGCGTTGAGATTGTTCA	-3'	
Cat	Forward	5'-	CGACCAGGGCATCAAAAACTT	-3'	NM_009804
	Reverse	5'-	AACGTCCAGGACGGGTAATTG	-3'	
Actb	Forward	5'-	GCCTTCCTTCTTGGGTATGG	-3'	NM_007393
	Reverse	5'-	GTGTTGGCATAGAGGTCTTTACG	-3'	

Abbreviations: *Ppara* (PPARα), peroxisome proliferator activated receptor alpha; *Cpt2* (CPT2), carnitine palmitoyl-transferase 2; *Acadvl* (VLCAD), very long-chain acyl-CoA dehydrogenase; *Acadm* (MCAD), medium-chain acyl-CoA dehydrogenase; *Hadha* (TPα), mitochondrial trifunctional protein α subunits; *Acox1* (ACOX), acyl-CoA oxidase; *Ehhadh* (PH), L-peroxisomal bifunctional protein; *Acaa1* (PT), peroxisomal 3-ketoacyl-CoA thiolase; *Sod1* (SOD1), superoxide dismutase 1; *Sod2* (SOD2), superoxide dismutase 2; *Cat* (Catalase); *Actb* (β-actin).
